# A Rare Case of Acute Necrotizing Encephalopathy of Childhood: A Case Report

**DOI:** 10.7759/cureus.55780

**Published:** 2024-03-08

**Authors:** Kushal K Bothara, Aryaman Dhande, Suhas M, Prajakta Kirdat Patil

**Affiliations:** 1 Radiodiagnosis, Dr. D. Y. Patil Medical College, Hospital & Research Centre, Dr. D. Y. Patil Vidyapeeth, Pune, IND

**Keywords:** acute necrotizing encephalopathy, acute necrotizing encephalopathy of childhood, dengue complication, neuroradiology, para-infectious condition, paediatric neurology

## Abstract

Acute necrotizing encephalopathy of childhood (ANEC) is a severe neurological disorder characterized by rapid-onset encephalopathy, often associated with viral infections. Acute necrotizing encephalopathy of childhood is associated with a very high mortality rate, and survivors may face long-term neurological sequelae. Acute necrotizing encephalopathy of childhood needs to be differentiated from its closest differential diagnosis, acute disseminated encephalomyelitis (ADEM). Most of the patients with ADEM recover, with a few of them having residual neurological deficits. We present a case of an eight-year-old boy with an acute history of fever, febrile seizures, and drowsiness. Magnetic resonance imaging revealed a symmetric tricolor appearance of bilateral thalamic lesions, characteristic of ANEC.

## Introduction

Acute necrotizing encephalopathy of childhood (ANEC) is a specific type of encephalopathy triggered by viral infections and caused by the focal breakdown of the blood-brain barrier [1]. This disorder is typically para-infectious, exhibiting limited signs of inflammation. Acute necrotizing encephalopathy of childhood primarily affects children and infants, leading to multiple symmetrical lesions in the thalami, putamina, white matter of the cerebral and cerebellar cortex, and the brain stem [2]. The disease progresses rapidly, marked by a sudden onset of seizures, altered consciousness, and vomiting, carrying a high risk of mortality and severe neurological complications. Pathologically, evident signs include swelling, petechial hemorrhaging, and tissue necrosis. Thorough clinical, radiological, and pathological evaluation, coupled with the exclusion of similar conditions, aids in diagnosis [2,3]. With no defined treatment or preventive measures determined for this condition, only 10% of the patients show complete recovery [4]. Here, we present a case of an eight-year-old patient with ANEC.

## Case presentation

An eight-year-old male patient, a known case of global developmental delay with a history of febrile seizures and not on anti-epileptics, presented to the casualty with complaints of severe dehydration and low-grade fever for two days. The fever was not associated with chills and rigors and had subsided with medications. The patient also reported vomiting, loose stools, and drowsiness over the last 24 hours, with increased drowsiness since the day of the visit. Additionally, the patient began experiencing seizures characterized by tonic movements of both upper and lower limbs and uprolling of the eyes. Each episode lasted for one to two minutes and was associated with postictal drowsiness. There was no evidence of a history of bowel or bladder incontinence. The patient had been on Cognicare syrup for three months.

The brain MRI report from 2022 suggested generalized cerebral atrophy. The patient had a normal birth history and was immunized up to the age of five. There was a history of third-degree consanguinity. The patient's second sibling (male) died at the age of two due to an acquired acute central nervous system (CNS) infection, accompanied by neuroregression and seizures.

In the casualty, the patient was continuously seizing and received two doses of intravenous (IV) midazolam along with IV fluids before being transferred to the pediatric intensive care unit (PICU). The patient exhibited microcephaly and dysmorphism. Despite an afebrile temperature, the respiratory rate and blood pressure were within normal ranges. Cardiovascular and respiratory examinations revealed no obvious abnormalities. The CNS examination indicated a low Glasgow score (E2V1M3), absent deep tendon reflexes, and a positive Babinski sign. The patient was placed on oxygen support and administered levetiracetam, phenytoin, IV adrenaline, and an IV packed cell transfusion.

Cerebrospinal fluid (CSF) examination revealed raised proteins (230.40) and glucose (138 mg/dl). On microscopic examination, confirmed wet mount findings were present on the Leishman stain. The patient was dengue NS1-positive.

Laboratory investigations tabulated in Table 1 revealed reduced hemoglobin, a reduced total leukocyte count, and increased blood urea levels.

**Table 1:** Laboratory parameters of the patient

Laboratory parameters	Patient's values
Hemoglobin	7.90 g/dL
Total leukocyte count	1700/ cumm
Platelets	262000/ cumm
Packed cell volume	27.20%
Mean cell volume	48.30 fL
Urea	61 mmol/L
Creatinine	0.54 mg/dL
Sodium	131 mmol/L
Potassium	3.71 mmol/L
Chloride	97 mmol/L
Albumin	4.10 g/dl
Globulin	3.60 g/dL
Albumin: globulin ratio	1.14
Prothrombin time (PT)	13.70 seconds
Activated partial thromboplastin time (aPTT)	20.80 seconds
International normalized ratio (INR)	1.15

The patient underwent an MRI of the brain, which revealed multiple symmetrically ill-defined T2-weighted images/fluid-attenuated inversion recovery (FLAIR) hyperintense areas in the bilateral cerebellar hemispheres. These areas predominantly involved the white matter, medulla, pons, midbrain, both thalami, posterior limb of the internal capsule, splenium, bilateral lentiform nucleus, and centrum semiovale. Marked diffusion restriction with corresponding low apparent diffusion coefficient (ADC) values was observed, along with multiple hemorrhagic foci showing blooming on susceptibility-weighted imaging (SWI). The bilateral thalami appeared edematous, exhibiting a tricolor appearance on ADC. Post-contrast images displayed mild peripheral enhancement in bilateral thalamic, pontine, and corona radiata lesions. The anterior portion of the third ventricle appeared slightly compressed, and both lateral ventricles showed mild dilatation. Generalized cerebral atrophy was also observed. Based on these findings, acute necrotizing encephalopathy (ANE) was considered more likely than acute disseminated encephalomyelitis (ADEM) due to the bilateral symmetrical distribution in deep gray matter nuclei and the cerebellum, along with the acute onset of symptoms.

**Figure 1:**
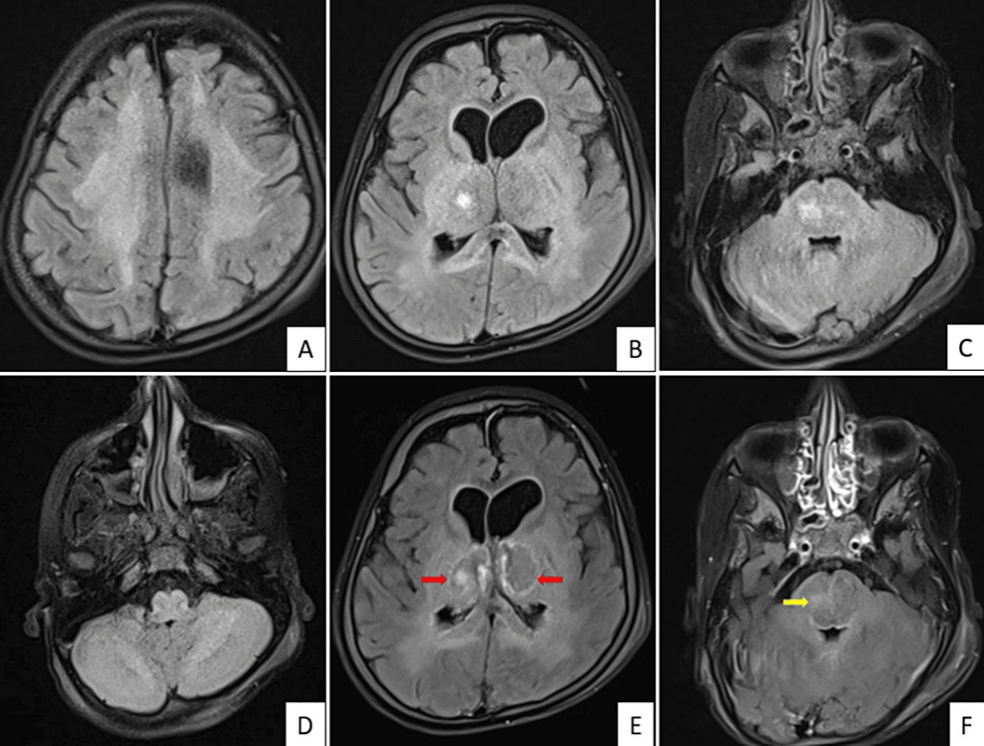
The FLAIR images (A–D) show bilateral diffuse symmetric hyperintense signal in the centrum semiovale (A), thalamus (B), pons (C), and cerebellum (D). Post-contrast FLAIR images (E, F) show peripheral contrast enhancement in the bilateral thalamus (red arrows) and subtle enhancement in the pons (yellow arrow). FLAIR: fluid-attenuated inversion recovery

Figure 1 shows FLAIR images (A-D) revealing bilateral symmetric areas of hyperintense signal in the centrum semiovale, thalamus, pons, and cerebellar hemispheres. Post-contrast FLAIR images (E-F) display peripheral enhancement in the bilateral thalamus and subtle enhancement in the pons.

**Figure 2:**
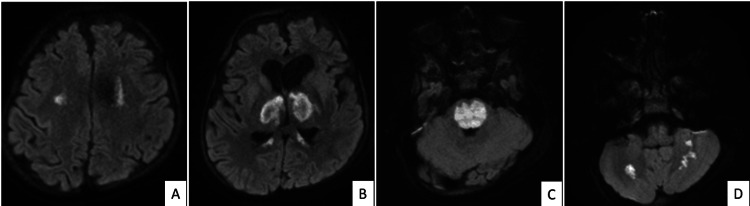
The DWIs (A–D) show bilateral symmetric areas of diffusion restriction in the centrum semiovale (A), thalamus (B), pons (C), and cerebellum (D). DWI: diffusion-weighted image

Figure 2 shows diffusion-weighted images (DWIs) (A-D) revealing areas of diffusion restriction in the bilateral centrum semiovale, thalamus, pons, and bilateral cerebellar hemispheres.

**Figure 3:**
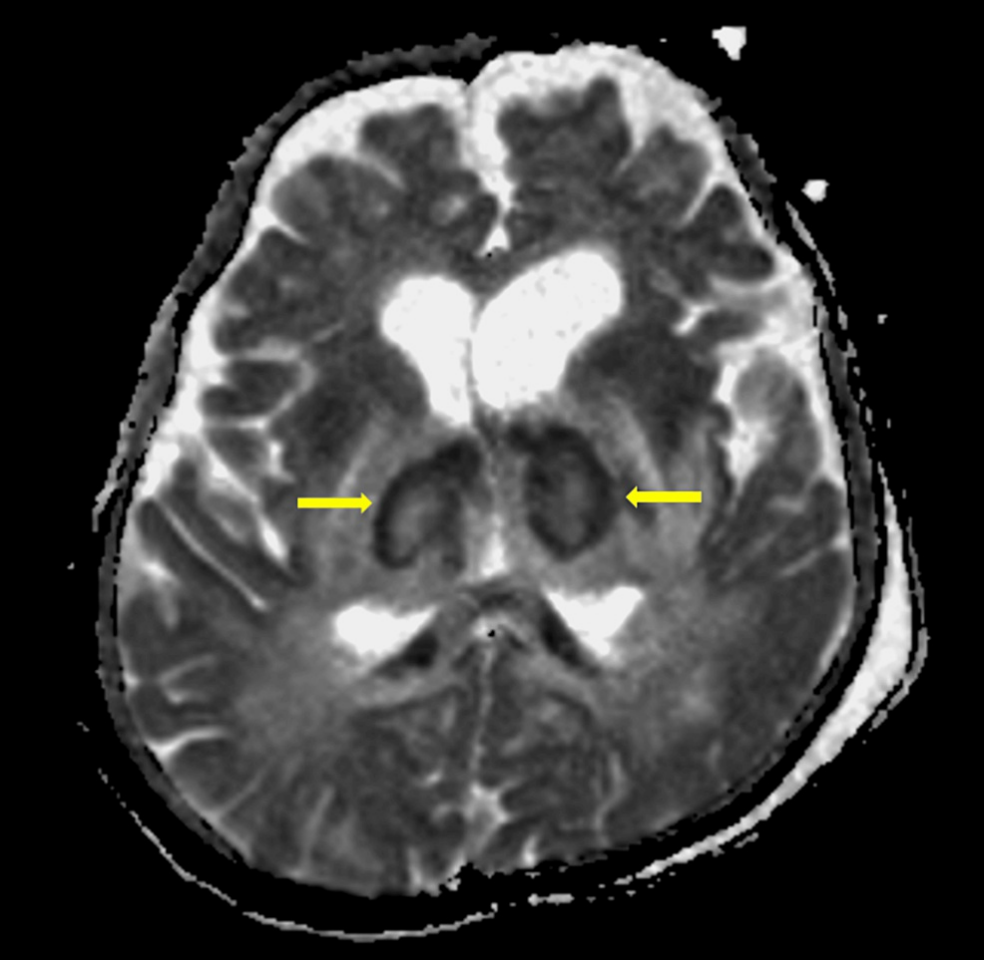
Tricolor appearance of bilateral thalami in ANEC The ADC image shows a tricolor appearance in bilateral thalami (yellow arrows). ANEC: acute necrotizing encephalopathy of childhood; ADC: apparent diffusion coefficient

Figure 3 depicts an ADC image showing a tricolor appearance of bilateral thalami.

**Figure 4:**
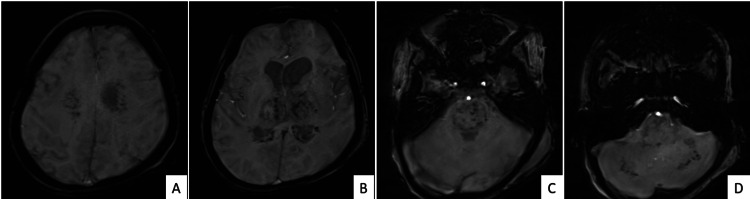
The SWIs (A–D) show symmetric areas of blooming in the centrum semiovale (A), thalamus (B), pons (C), and cerebellum (D). SWIs: susceptibility-weighted images

Figure 4 shows SWIs (A-D) revealing areas of blooming in bilateral centrum semiovale, thalamus, pons, and bilateral cerebellar hemispheres. 

Despite the administration of supportive therapy in the PICU, the patient's condition continued to deteriorate over the next few days, ultimately leading to his demise.

## Discussion

Acute necrotizing encephalopathy of childhood is a pauci-inflammatory and para-infectious complication associated with multiple viral infections, leading to blood-brain barrier breakdown due to intracranial cytokine storms. It typically does not cause direct viral CNS infections or demyelination [5]. In our case, the patient had a dengue infection. Cases of ANEC following dengue infection and other viruses such as coronavirus, influenza, and Japanese encephalitis virus in children have been documented by Kamble et al. and Kulkarni et al. [6,7].

The occurrence of ANEC has also been genetically linked, originating from a missense mutation in the RAN binding protein 2 (RANBP2) gene, categorized as a subtype of acute necrotizing encephalopathy known as ANE1. Positive family history often extends beyond first-degree connections. Instances of encephalopathy preceding the illness in the patient or their family members may not have been recognized as acute necrotizing encephalopathy but rather classified as ADEM, primary angiitis, cerebellitis, etc. [8].

The imaging features of ANEC include the presence of multiple symmetrical brain lesions affecting both gray and white matter, involving the thalami, brain stem, cerebral white matter, and cerebellum. Acute necrotizing encephalopathy of childhood is characterized by the bilateral involvement of the thalami, which presents as swelling and diffusion restriction on DWI. On ADC, bilateral thalamic lesions exhibit a tricolor appearance, with a central high signal, a peripheral low signal, and an outermost area of intermediate signal intensity [9].

The closest differential diagnosis for ANEC is ADEM. In ADEM, multiple T2/FLAIR hyperintense lesions with restricted diffusion can be observed, involving bilateral white and gray matter, including the thalami and brainstem. However, the spatial distribution in ADEM is typically asymmetrical, and spinal cord involvement is common. Clinically, symptoms are similar, such as fever, headache, and signs of encephalopathy. Nevertheless, the clinical course in ADEM tends to be more gradual compared to the rapid onset seen in ANEC [10]. Acute necrotizing encephalopathy of childhood represents a serious and often lethal complication, with prognosis varying from complete recovery to death within days of admission, irrespective of treatment.

## Conclusions

Acute necrotizing encephalopathy of childhood is a severe neurological condition primarily associated with viral infections, resulting in rapid-onset encephalopathy in children. The diagnosis of ANEC relies on clinical features, characterized by a very rapidly progressive and fulminant course, and radiological findings, including multiple symmetric lesions in the bilateral gray and white matter of the cerebrum and cerebellum, with a characteristic edematous and tricolor appearance of the bilateral thalami. Early detection and diagnosis of ANEC are facilitated by MRI and CSF examinations. Early recognition, prompt medical intervention, and supportive care are crucial in improving outcomes for individuals affected by ANEC. Continued research is essential to better understand the underlying mechanisms and develop targeted therapies for this complex condition.
